# The science and legacies of Ronald Phillips: A brief perspective

**DOI:** 10.1002/tpg2.70163

**Published:** 2025-12-09

**Authors:** Richard B. Flavell

**Affiliations:** ^1^ International Wheat Yield Partnership Durham North Carolina USA

## Abstract

Ronald Phillips, a maize geneticist, developed his career exploiting maize and the genetics of other species to help bring plant science into the era of molecular genetics. He was driven by belief in the value of service for the common good and in the value and importance of science for its own sake and for agriculture and food security, in particular. His career was a journey along the frontiers of plant science—from early DNA isolation to whole genome sequence revelations and into agricultural biotechnology. He represented the progress along the way in the maize genetics community, in national and international science and at the highest levels of influence. He was a caring, celebrated scientist who made a difference for people and institutions and left plant science so much further advanced than when he joined it in the mid‐1960s.

## Plain Language Summary

This is a brief appreciation of Ronald Phillips, a maize geneticist, who during his career made noteworthy contributions to plant science and agricultural biotechnology. Some of his salient contributions and legacies are summarized.

1


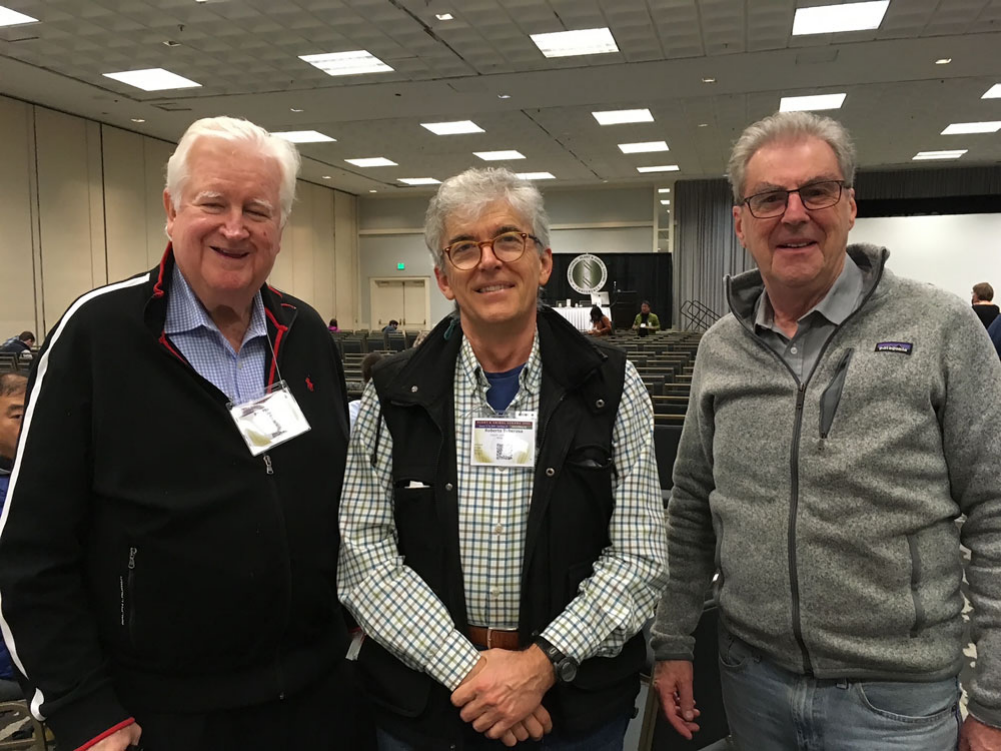
 Ronald Phillips, Roberto Tuberosa, and Richard Flavell. Photograph taken in 2018 at the Plant and Animal Genome Conference (PAG 25), San Diego.

## 2 OVERVIEW

Scientists often wonder about the value of their contributions to science, technology, and society and their resulting legacy. Is it the published papers, the talks in conferences or university classrooms, the positions of office held, the help given to students and postdocs, or reviews and chapters written that have had the most significant impacts? Often the answers remain unclear because there were no means of tracking the impacts of scientific influencers. While the answer varies for each of us, for Ronald Phillips many or all of the items in the above list are relevant. This is because all the items in this list were important to him and he was a very active participant, servant, and leader of science wherever it took him.

It is right and proper that influential scientists are recognized and remembered. They also need to be appreciated in the context in which they lived, worked, and thought. This seems even more important as old issues of journals are not on library shelves, science becomes more short‐term in outlook and there is, rightly, greater emphasis on looking forward than backward.

Ron was born in 1940, before there was a formal proof that DNA embedded in chromosomes was the molecule carrying the code of heredity. (It was only in 1944 that the much‐quoted paper from Avery, MacCleod, and McCarty (1944) was published that provided proof that DNA was the heredity material.) Thus, Ron's life, like mine, spanned the period of DNA becoming proven as the basis of heredity in plants to the present where whole genomes are sequenced with ease and accuracy. He gained his bachelor's degree in 1961 and a master of science in 1963 from Purdue University, Indiana. His PhD was gained from the University of Minnesota in 1966, and he was appointed to a faculty position there in 1967 where he stayed for the remainder of his career. However, during the year before his appointment at the University of Minnesota, he was an National Institutes of Health‐supported trainee working under Adrian Srb at Cornell University. There he was able to show that linkage group V of *Neurospora crassa* was chromosome 2 and also identified a new ascospore mutant. Thus he, like me, focused on Neurospora in the mid‐1960s. This species had become a model fungus, alongside yeast, for genetic studies where “one gene‐one enzyme” hypotheses were being explored and mutants mapped to linkage groups and chromosomes through straightforward Mendelian genetics. The more advanced fungal genetics surely left its mark on his thinking.

By the late 1960s, that is, soon after his appointment at the University of Minnesota, he was already publishing papers on chromosome interchanges, gene mapping and recombination in maize, having been influenced (and taught) by the expert maize geneticist Charles Burnham at the University of Minnesota whom he greatly admired. No doubt it was satisfying and appropriate to him to be focusing on maize having been brought up on a farm in Indiana and therefore being fully familiar with the crop in the field. By 1970, Ron had committed himself to being a maize geneticist, making deep commitments to its chromosomes and recognizing its great assets for genetics and crop biology. Maize had already become a leading plant for studying genetics in the United States, in part through the revealing studies of the pioneers of genetics during the first half of the 20th century. Ron's early focus on whole chromosomes (through cytology and genetics) remained a theme throughout his career. Maize genetics also remained central even though he championed the genetics of other plant species as he emerged as a leader/spokesperson for plant genetics/genomics as a whole.

Like all scientists, Ron had to grow from being a young researcher with limited confidence and experience to assimilate masses of new knowledge, as part of his path to scientific maturity. Readers should remind themselves that he started his career as an experimental scientist when little was known about the structure and informational content of chromosomes. It was before DNA sequencing, recombinant DNA, cloning, restriction enzymes, and polymerase chain reactions were in use, and before characterization of indels, single nucleotide polymorphisms, restriction fragment length polymorphisms, simple sequence repeats, and amplified fragment length polymorphisms, that is, basic tools in genetics today. It was before Arabidopsis and rice were developed as models to overcome the constraints of maize and other plant species that hindered their wide adoption as experimental organisms.

At the start of his career, he did not know that the maize chromosomes his trained eye looked at in his microscope consisted mostly of repeated sequences and not genes and had therefore evolved by a plethora of amplification events. Neither was he aware that plant chromosomes were full of transposons, which could move and have effects on neighboring genes, in spite of McClintock (1956) having revealed examples of this. Nothing was known about transcription factors, non‐coding DNA, siRNAs, non‐coding RNAs, and epigenetics. There were no plant gene or genome sequences, or databases, or even desktop computers to help with personal research projects.

During the 1970s there was an explosion in the knowledge and understanding of genetics coming from organisms other than plants, including Drosophila, yeast, other fungi, man, and bacteria that needed to be assimilated, filtered, and assessed in terms of their potential relevance to maize. All this meant that Ron, like others, needed to think, listen, read, discuss, wonder, and make choices about what to explore in maize, using its comparative advantages. His scoping of what was possible was achieved by attending lots of meetings and reading and listening widely. He then picked carefully what could be explored in corn. A serious approach was required, but he was the kind of person who took things seriously.

Over the years he assimilated the knowledge, tools, and techniques of modern genetics, including molecular biology, as they emerged, to address topics in maize genetics/cytogenetics that interested him. Some of the topics were clearly chosen because of their greater relevance/potential to practical agriculture. The topics to which he devoted most attention in his own practical research included the structure of the nucleolus organizer region; DNA endoreduplication; use of duplicate‐deficient chromosomes; male sterility; aneuploid syndromes; maize tissue culture and regeneration; tissue culture‐induced variation; grain quality; oat‐maize addition lines; and the genetics of flowering time, including the positional cloning of a major quantitative trait locus (QTL) controlled by a non‐coding sequence (Salvi et al., 2007). The results of his team and their research publications became the vehicles for ramping up his understanding, university teaching, talks to external audiences, and societies where impact was generated through the word of mouth, discussion, and personality. As a result, he became well known for bridging maize genetics/cytogenetics, biotechnology, and agriculture across public and private sector research.

In this perspective, rather than reviewing all these research topics and outputs (such a text was produced on his formal retirement in 2009; Kaeppler et al., 2009), I have selected four areas that highlight the major packages of science in his publication list to illustrate his research career and applications/approaches to maize genetics/cytogenetics.

## 3 RESEARCH TOPIC 1: NUCLEOLAR ORGANIZERS AND DIFFERENTIAL GENE EXPRESSION

As methods to investigate the molecular biology of chromosomes started to gather pace in the 1970s, the ribosomal RNA (rRNA) genes clustered at nucleoli became one of the first to be studied because of their high copy number and therefore ease of recognition by DNA/RNA hybridization. The sites where nucleoli formed (nucleolar organizer regions [NORs]) are visible as constrictions in metaphase chromosomes. Ron had noted a cytogenetically deviant strain that had two nucleolar organizers, and this strain enabled him, Kleese and Wang, using DNA‐rRNA hybridization methods, to conclude that the nucleolus organizer was the site of rRNA genes, as predicted from other species (Phillips et al., 1971). They claimed this was the first time proof of the location of rRNA genes at the nucleolus organizer was reported for plants. It was possible later to carry out in situ hybridization of radioactively labeled RNA to metaphase chromosome spreads and indeed see the sites of hybridization, thereby proving the sites of rRNA gene clusters in chromosomes. Examination of these chromosomal hybridization sites by Ron and his team also revealed that most of the rRNA genes lay outside the nucleolus, at sites of heterochromatin as had been shown by others in other species (Givens & Phillips, 1976). This emphasized the conclusion that there was an excess of rRNA genes and not all were used/transcribed, at least in most cells. Further work by the team showed, as had others in other species, that there was substantial variation in the numbers of rRNA genes at different maize NORs and in different inbred lines.

Some NORs in maize are relatively silent with few genes being transcribed, and the team showed, as had others in other species, that moving NORs into different genetic backgrounds in crossing programs sometimes leads to silencing of NORs (Givens & Phillips, 1976). This illustrated the phenomenon of “nucleolar dominance.” Ron's team also found that chromosome rearrangements such as translocations, deletions, and duplications can dramatically affect nucleolar size and activity. When genes were cloned, restriction mapped, and subsequently sequenced, the team was able to determine the structure of the rRNA gene repeats, revealing similarities in structure with those of related plant species (McMullen et al., 1986). The role of genetic variation in rRNA genes in agricultural productivity remains unknown, and the precise molecular basis of nucleolar dominance is still incompletely understood.

Ron and his team published numerous papers on the nucleolus and rRNA gene system during the 1970s–1980s when this topic was current. The same techniques enabled the team to also define the chromosomal sites of the ribosomal 5S RNA sequences. This work on NORs is noteworthy because it illustrates that Ron and his team sought to bridge cytogenetics and molecular biology from the earliest days of plant molecular biology, indeed before there was a subject named plant molecular biology.

## 4 RESEARCH TOPIC 2: MAIZE TISSUE CULTURE AND REGENERATION OF FERTILE PLANTS

In the early 1970s, when tissue culture became a significant part of dicot plant biology, Ed Green started a maize tissue culture program at the University of Minnesota. There were numerous goals, including the potential to select mutants in culture that were more difficult to select in differentiated plants. Callus was readily induced and maintained from scutellum tissue of immature embryos of various hybrids and inbreds using 2,4‐dichlorphenoxyacetic acid (2,4‐D). The calli were readily propagated and maintained on media containing 2,4‐D. Plantlets emerged by callus differentiation, and whole plants were achieved by manipulation of the hormone levels. This was published in 1975 and was the first publication to provide a detailed recipe for maize plant regeneration from tissue cultures (Green & Phillips, 1975). Importantly, genetic variability for callus growth, differentiation, and plant regeneration was observed among both hybrids from single crosses and inbreds. This emphasized that there was a strong genetic component to callus growth in tissue culture and regeneration as later detailed with the identification of QTLs influencing callus regeneration summarized by Armstrong et al. (1992).

The collaboration between Ed Green and Ron Phillips started to explore this variation. Large numbers of genotypes were surveyed using their culture and regeneration recipes. The strain A188 was found to be particularly responsive to the culturing conditions adopted. This contrasted with most other maize strains, and so A188 became the standard lab strain for maize tissue culture and regeneration in the maize community, thereby making the contributions of the Minnesota group's findings particularly noteworthy and useful.

The plants they regenerated were readily seen to be variant, displaying a different array of traits from the starting accession. This demonstrated for maize the concept of “somaclonal variation,” previously recognized in dicot cultures. To a geneticist like Ron there were the questions of how, when, and why the variation originated. To him, the cytogeneticist, looking at chromosomes was an obvious approach. A high frequency of chromosome abnormalities was seen, including polyploidy, aneuploidy, and broken chromosomes. Subsequent studies revealed numerous sources of mutations originating from chromosome break‐fusion bridge cycles, well recognized and understood by cytogeneticist Phillips (Armstrong & Phillips, 1988). Subsequent analyses of plants from the first and second self‐pollinated generations revealed at least 50 observable phenotypes segregating as if controlled by single recessive mutations. The frequency was greater when regeneration occurred from older cultures. Chimeric plants were also found, and it was concluded that some mutations occurred in sectors of the plants during plant regeneration. All these observations prompted comparisons with the instabilities described by McClintock earlier and her conclusions regarding the innate susceptibilities of chromosomes to change (McClintock, 1950).

Later, when transposable elements had been purified by molecular cloning and it became possible to assess the activity of such elements, activation of Ac and Spm elements was found to be associated with tissue culture, echoing the conclusions of Barbara McClintock that when (genetic) shocks are experienced by maize cells, transposable elements become more active (Peschke et al., 1987). Furthermore, when activation of genes had been found to be correlated with changes in cytosine methylation, the Minnesota team showed that passage of maize cells through tissue culture and regeneration was found to lead to many changes in DNA cytosine methylation (Kaeppler & Phillips, 1993).

## 5 TISSUE CULTURE AND FERTILE PLANT REGENERATION AS STEPPING STONES TO AGRICULTURAL BIOTECHNOLOGY

To enable new genes to be introduced into plants, it was recognized early on that regeneration of a whole fertile plant from a transformed cell, selectable in culture, was necessary. Thus, the culture and selection of totipotent cells and their regeneration into fertile plants became the basis of agricultural biotechnology, maize included. Much of this was built upon the progress of Green and Phillips in defining and enabling somatic embryogenic cultures and their regeneration into fertile plants. The first transgenic maize plants were created from such somatic embryogenic culture procedures (SC82 and SC719) using the gene gun to introduce the selectable Bar gene. The A188 strain, and hybrids with it, remained in extensive use in academia, following the studies of Green and Phillips, and it was the first maize line to be transformed by the addition of new genes using *Agrobacterium tumefaciens* (Ishida et al., 1996). However, this strain was completely inadequate for commercial applications where elite lines fit for agriculture needed to be genetically engineered. The presence of mutations arising during the tissue culture and regeneration needed for *Agrobacterium tumefaciens* transformation was another major hurdle for the birth of maize biotechnology. The major corn companies of Monsanto and Pioneer Hybrid therefore needed to find different recipes to create agriculture‐compatible genetic engineering approaches for elite germplasm. Their successes in overcoming these issues for a range of elite maize inbreds enabled them to lead the field of maize agricultural biotechnology for decades. The discovery of A188's efficiencies in somatic embryogenesis and the associated developments were indeed catalytic for the birth of maize genetic engineering but inadequate for commercial deployment per se.

Genetic analyses of the regenerants in A188, through crossing programs, revealed that the trait of prolific embryogenesis and regeneration was genetically complex, under the control of multiple QTLs, and other lines could be improved in regenerability by stacking multiple QTLs from A188 (Lowe et al., 2006).

The Minnesota plant genetics/biotechnology team was part of a new company, “Molecular Genetics Inc.,” in the 1980s and its successor, “Biothink,” and Ron was an active participant and Science Board member. This was the period when numerous new plant science companies were initiated in recognition of the new possibilities emerging from plant science, although Molecular Genetics Inc. spanned both plant and human health applications.

## 6 RESEARCH TOPIC 3: GENETIC MAPPING OF MAIZE CHROMOSOMES USING OATS X MAIZE CROSSES

In the early 1990s Ron teamed up with Howard Rines in the University of Minnesota to advance the genetics of hexaploid oats and, as it turned out, to also progress the genetic mapping of maize. The program included the making of oats × maize hybrids. This followed the model production of wheat haploids gained by crossing hexaploid wheat × maize plants in which it was found that the maize chromosomes became eliminated during meiosis leaving a haploid set of wheat chromosomes (Laurie & Bennett, 1988). Subsequent culturing of the immature embryos enabled the rescue of haploid plants, which could be converted into hexaploid plants by culturing with colchicine. This wide cross approach was therefore a very rapid and useful way of making haploids for breeding and genetic studies. However, making such crosses between oats and maize and generating progeny was far from easy. Rines and Phillips finally achieved success, and among the progeny were occasional lines with a complete hexaploid set of oat chromosomes and a disomic pair of maize chromosomes (Ananiev et al., 1997). In this way the Rines/Phillips team succeeded in making a set of lines that had disomic additions of individual maize chromosomes 1–9 and part of chromosome 10 (Kynast et al., 2001). The availability of stable lines of oats with a disomic pair of a maize chromosome enabled maize markers (and genes) to be mapped to individual maize chromosomes without the need for segregating populations (Ananiev et al., 1997). This approach also enabled mapping of paralogs to different chromosomes, something not readily achieved in those days for duplicated genes in their native genetic background.

Rines and Phillips took the approach further by adopting the techniques of radiation mapping used extensively in animal cell genetics. Treatments of cells with X‐rays lead to chromosome breaks and only chromosome pieces with centromeres are thus able to replicate and divide regularly and survive. After treating the maize disomic addition lines with X‐rays, they searched for lines with smaller fragments of maize chromosomes that survived the radiation treatments. The use of marker technologies on these lines enabled more precise mapping of centromeres, markers, genes, and QTLs to chromosome segments and establishment of linkage maps. Numerous papers were published on the mapping of markers on maize chromosomes based on the use of oats/maize addition lines (e.g., Kynast et al., 2004; Okagaki et al., 2001). These added some utility until genome sequencing, achieved by others, enabled comprehensive mapping of markers across chromosomal maps. The oats × maize experiments showed the motivation of Phillips to adopt tricks of cytogenetics to advance chromosome mapping. The approach of making wide crosses also led to the production of haploid oats lines, which could be doubled to make fertile oats haploids, as had been done with wide crosses with wheat. Thus, the Minnesota work also enabled the making of haploid oats, useful in oat breeding (Rines, 2003).

## 7 RESEARCH TOPICS 4: HETEROCHROMATIC KNOBS IN MAIZE CHROMOSOMES

Cytologically observable heterochromatic components of (pachytene) chromosomes were described by McClintock in 1929 and were well known to Phillips. The number, size, and position of heterochromatic knobs vary between different maize lines and are found in 23 different positions in the 10 maize chromosomes. As genome sequencing emerged, Phillips returned to his cytogenetics to address the physical and genetic mapping of heterochromatic chromosome segments. Again, the single maize chromosome addition lines in an oats genetic background enabled the structure of defined maize heterochromatic segments to be explored one at a time (Ananiev et al., 1998). The structures of knobs comprised tandem arrays of 180 bp repeats, and the same 180 bp repeats were found in different knobs, but not centromeres or NORs, although the copy number varied enormously. The Minnesota team found that the arrays are interrupted by insertions of transposable, mostly full length, elements, and there are preferred hotspots for insertion of such elements. These sorts of features were typical of heterochromatic segments in other species. Such studies nicely enriched the findings from the early era of maize cytogenetics with the emerging techniques of chromosome molecular biology.

As noted above, there were many more topics studied by Phillips and his teams giving him a broad profile of discovery within maize and other plant species. These can be seen from his publications noted in search engines.

## 8 THE IMPACTS AND LEGACIES OF RON PHILLIPS

The catalogue of activities and outputs of Ron Phillips over his career is exceptionally noteworthy. Many of us are scientifically busy, attend scientific meetings to learn, meet colleagues, and give presentations and are invited here and there to give seminars—this is the life of a successful scientist in academia. But the number of inputs and high‐profile outputs from Ron Phillips’ scientific life are extraordinary. His writings of journal articles, book chapters, abstracts, and proceedings of meetings numbered some 575 over his 40‐year career. He gave over 350 invited seminars at universities, companies, and scientific symposia. As a university professor, he taught courses, inspiring and shepherding over 50 students through their master's or PhD degree courses and mentoring over 20 postdoctoral scientists as well as many visiting scientists to his laboratory. Obviously, through all these activities and outputs, he taught and influenced many thousands of scientists, in addition to all those he taught at the University of Minnesota.

In response to all these outputs of contributions to scientific society and his deep yet broad knowledge that he could articulate, he was elected President of the Science Society of America (1999–2000) and Chair of the Council of Scientific Society Presidents (2006). For his contributions to plant genetics and especially to biotechnology he was elected as a Member of the National Academy of the United States of America in 1991. He was also awarded an honorary doctorate from Purdue University from which he had gained his bachelor's and master's degrees. He was also offered many positions of leadership, responsibility, and acclaim that required mature judgments, wisdom, and decision‐making. Among these were his leadership in USDA as Program Director in the Competitive Research Grants Office (in 1979) and as Chief Scientist in the National Research Initiative 1996–1998 during which time he chaired the White House Committee where the Plant Genome Research Initiative was formulated. Not surprisingly, he was awarded several noteworthy prizes, including the Wolf Prize for Agriculture given by the Wolf Foundation in Israel. This latter award is often described as a Nobel Prize for Agriculture. In 2010 he received the ISA Medal for Science from the University of Bologna and the Siehl Prize from the University of Minnesota. Recognition also came by being invited to serve on scores of national and international boards, committees, and panels to advise and to oversee the running of important large institutions such as the International Rice Research Institute in the Philippines.

While highlighting Ron's activities and outputs, I want to emphasize that while he was creating them and serving roles in the United States and abroad, he was not only giving of his experience to others but also learning. Such learnings could then be used in the next activity. Each activity therefore served as a rachet to enhance his experience, wisdom, and foresight substantially above those of most scientists. In my opinion, his catalogue of primary research publications is not extraordinary in number or impact, but his use of scientific research outputs to fit him for more and better public service is very noteworthy and outstanding. These learnings without doubt made “the whole” of his scientific contribution very much more than “the sum of the parts.”

His chosen scientific life was not easy. It required long hours of work, punishing travel schedules, and an intensely cluttered diary, all of which no doubt generated stress. It required the support of those around him. Why did he grow this kind of career? The science we do reflects who we are, our interests, goals, and more importantly, our beliefs, passion, and senses of commitment. Ron was driven by his belief in the value of service for the common good and his beliefs in the value and importance of science for its own sake and for agriculture and food security, in particular. I also know he enjoyed his career, intellectually and through the friendships he made *en route*.

In summary, Ron was greatly influenced by early exposure to maize cytogenetics and carried the torch for this, with humility, through the molecular genetics era. His career took a journey along the frontiers of plant science—from early DNA isolation to whole genome sequence revelations and into agricultural biotechnology. He followed it all in detail and represented the progress along the way in the maize genetics community, in national and international science, and at the highest levels of influence. The results generated within his research program with students and postdoctoral fellows served as vehicles to drive forward his visions of plant genetics, nationally and internationally.

The largest part of Ron's legacy was built from the diversity of impacts he generated in his very extensive mentorship and teaching of others at all levels. He was a man who was always prepared to give back to society, recognizing that public funds enabled him and most academic scientists to practice their art and move forward new options for science and society. He was a caring scientist who positively impacted people and institutions as he helped add the “Gene Revolution” to the “Green Revolution.” Clearly, Ron left plant science so much further advanced than when he joined it in the mid‐1960s (Kaeppler & Springer, 2024).


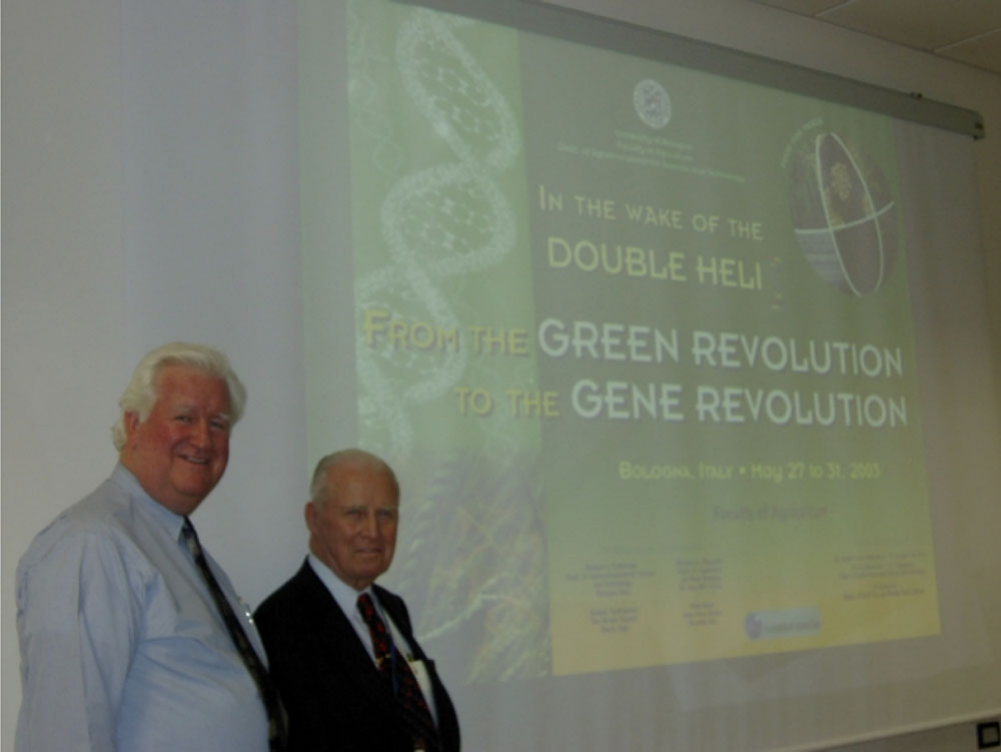
 Ronald Phillips and Norman Borlaug. Photograph taken in 2003 at the Congress “In the Wake of the Double Helix: From the Green Revolution to the Gene Revolution” held in Bologna, Italy.

## AUTHOR CONTRIBUTIONS

**Richard B. Flavell**: Conceptualization; formal analysis; investigation; project administration; validation; writing—original draft; writing—review and editing.
